# Dietary Vitamin C and Vitamin C Derived from Vegetables Are Inversely Associated with the Risk of Depressive Symptoms among the General Population

**DOI:** 10.3390/antiox10121984

**Published:** 2021-12-13

**Authors:** Anni Wang, Jia Luo, Tianhao Zhang, Dongfeng Zhang

**Affiliations:** Department of Epidemiology and Health Statistics, The School of Public Health of Qingdao University, 308 Ningxia Road, Qingdao 266071, China; 2020021077@qdu.edu.cn (A.W.); luojia@qdu.edu.cn (J.L.); 2020021075@qdu.edu.cn (T.Z.)

**Keywords:** vitamin C, depression, National Health and Nutrition Examination, cross-sectional study

## Abstract

Vitamin C is a water-soluble antioxidant. Reducing the level of oxidative stress can alleviate depression. Therefore, we investigated the correlation between dietary vitamin C intake and the risk of depressive symptoms in the general population. Data from the 2007–2018 National Health and Nutrition Examination Survey were used in our study. The dietary intake of vitamin C was assessed by two 24-h dietary recalls. Depressive symptoms were assessed with the Patient Health Questionnaire-9. Logistic regression and restricted cubic spline models were applied to assess the relationship between dietary vitamin C intake and the risk of depressive symptoms. The multivariate adjusted odds ratio (95% confidence interval) of depressive symptoms for the highest vs. lowest category of dietary vitamin C intake and vitamin C intake derived from vegetables were 0.73 (0.58–0.91) and 0.73 (0.56–0.95). In subgroup analyses, dietary vitamin C intake was negatively correlated with the risk of depressive symptoms in females 18–39 years old and 40–59 year-old groups. A dose-response analysis showed that there was a nonlinear relationship between dietary vitamin C intake and the risk of depressive symptoms. Dietary vitamin C intake and vitamin C intake derived from vegetables were inversely associated with the risk of depressive symptoms among the general population. We recommend increasing the intake of vegetables in daily diet.

## 1. Introduction

Depression might be related to a variety of factors, such as heredity [1], environment [2], and diet. According to previous research, there might be a negative correlation between depressive symptoms and the intake of nutrients, such as protein [3], carotenoids [4], fiber [5], natural folic acid [6], magnesium [7], and total zinc, iron, copper, and selenium intake [8]. Previous studies have shown that oxidative stress level in patients with depression is increased [9]. Vitamin C is a water-soluble antioxidant [10]. Vitamin C is also a cofactor of several important hydroxylation reactions in the human body, such as the synthesis of catecholamine [11,12]. The increase or decrease in catecholamine and other substances may be related to depression [13]. Some studies investigated the relationship between vitamin C and depression.

A previous study among male students aged 18–35 in New Zealand [14] showed that vitamin C levels were negatively correlated with depression. Another study [15] found that the intake of vitamin C in Japanese elderly people’s diet was negatively correlated with the risk of depression symptoms. It was also found that vitamin C supplementation was significantly related to a decreased depression score in depressed shift workers in an oil refinery [16]. However, a study on the elderly Merseyside residents over 60 years of age living in residential homes [17] did not find the association between vitamin C supplementation and the improvement of depressive symptoms.

Ascorbic acid mainly depends on the dietary intake of vegetables and fruits. Rare studies focused on the connection between vitamin C intake and the risk of depressive symptoms in the general population. At the same time, their dose-response relationship was also unclear. Therefore, we investigated the relationships between vitamin C (from dietary or different sources) and the risk of depressive symptoms in the general population.

## 2. Materials and Methods

### 2.1. Study Population

A National Health and Nutrition Examination Survey (NHANES) was approved by the National Health Organization Institutional Review Board, which is a nationally representative study, and all participants signed the informed consent [18].

**Figure 1:**
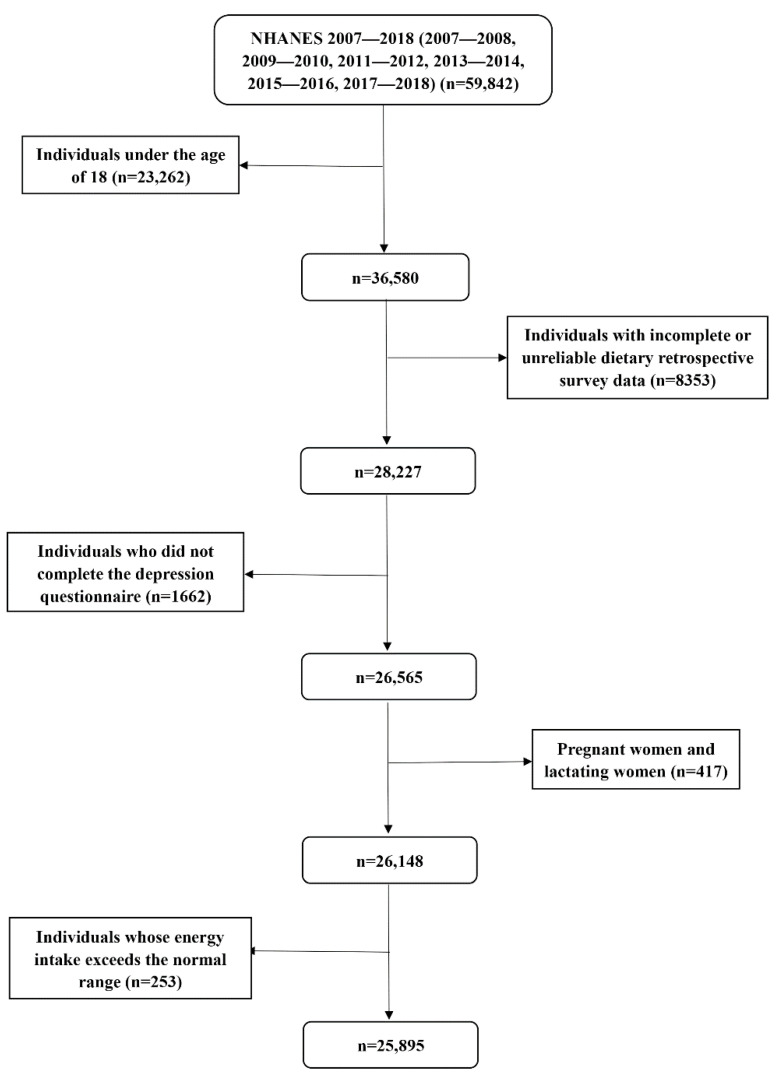
Flow chart of the screening process for the selection of eligible participants from NHANES 2007–2018.

This study analyzed the data of six investigation cycles, including 59,842 respondents. Individuals younger than 18 years old with incomplete or unreliable dietary retrospective survey data and incomplete depression questionnaire data were excluded. We further excluded pregnant females, lactating females, and individuals with extreme total energy intake (more than mean ± 3 standard deviations) [19]; participants under the age of 18 did not have complete data on depressive symptoms, and pregnant or lactating females might have special dietary intake and metabolism [20]. Finally, a total of 25,895 individuals were included in this study. The specific process was shown in Figure 1.

Among 25,895 individuals, 18,341 had completed data on vitamin C from vegetable sources, 10,700 had completed vitamin C data from fruit sources, and 8132 were users of vitamin C supplements.

### 2.2. Assessment of Depressive Symptoms

The outcome of the interest variable was depressive symptoms. The Patient Health Questionnaire (PHQ-9) is a nine-item scale with each item ranging from 0 to 3 [21]. We used it to evaluate the group of depressive symptoms. A total score ranged from 0 to 27, and 10 was the cut-off value [22]. According to the cut-off value, participants were divided into the depressive symptoms group and the non-depressive symptoms group.

### 2.3. Evaluation of Dietary Vitamin C Intake and Vitamin C Supplements

The daily vitamin C intake and vitamin C supplements of each individual was the average of two 24-h dietary retrospective interviews [23]. Vegetable and fruit sources of vitamin C were identified according to US Department of Agriculture (USDA) food codes from the Individual Foods data in NHANES [24]. Dietary vitamin C intake, different sources of vitamin C, and total vitamin C intake (food + supplements) were divided into 3 groups (T1, T2, and T3) according to the terciles. T1, T2, and T3 represented low, medium, and high vitamin C intake levels, respectively.

### 2.4. Covariates

According to previous literature on dietary intake and depression [25,26,27], we included a series of covariates. Demographic characteristic variables included sex, age, educational levels, marital status, poverty-income ratio (PIR), and race. Body mass index (BMI) was divided into three categories [28]. We also included some health behavior variables, such as alcohol consumption status and so on. Health factors included hypertension, diabetes, and stroke. In addition, we also adjusted the total energy intake [26]. Supplementary Table S1 describes the detailed condition of the covariates.

### 2.5. Statistical Analysis

In this study, we used Stata 15.0 (Stata Corp., College Station, TX, USA) for the main statistical analyses. According to the NHANES guidelines, we recalculated the new sample weights when merging two or more cycles of data [29].

We compared the characteristics with or without the depressive symptoms group and high or low vitamin C intake from vegetables. Characteristics of the study population were presented as numbers (percentages) for categorical variables. For continuous variables, we used the Kolmogorov–Smirnov normality test. If distribution conformed to the normal distribution, we used mean ± standard deviation (SD) to express it; otherwise, we used the median (interquartile range). If the distribution conformed to the normal, Student’s t-tests would be applied; otherwise, we chose the Mann-Whitney U test for comparison. We used the χ^2^ test for the classified variables.

The lowest vitamin C intake group (T1) was taken as a reference group. The connection between dietary vitamin C intake, vitamin C derived from vegetables and fruits, total vitamin C intake (food + supplements), and depressive symptoms risk were analyzed by the logistic regression model, and the results were reported as an odds ratio (OR). In addition, we divided the participants into two categories according to whether they were vitamin C supplement users or not.

Model 1 adjusted for age and sex. Model 2 additionally adjusted for education level, marital status, PIR, race, smoking, drinking, occupational and recreational physical activity level, BMI, hypertension, stroke, diabetes, and dietary energy intake. In addition, due to the relatively long time span, we added the time dummy variable into the regression model, and also increased the interaction of time dummy variables and vitamin C intake.

We performed sensitivity analysis by excluding participants\taking antidepressant medication (Bupropion, Fluoxetine, Sertraline, Paroxetine, Venlafaxine, Citalopram, Escitalopram, and Duloxetine). Considering different sex and age teams had different depression prevalence, we performed stratification analyses by sex and age. The dose-response relationship between dietary vitamin C intake and the risk of depressive symptoms was evaluated by a restricted cubic spline model, in which three nodes were located in the 5th, 50th, and 95th percentiles of dietary vitamin C intake. The result was statistically significant when the two-sided *p* value was less than 0.05.

## 3. Results

### 3.1. Characteristics of the Participants

As for the intake data, a total of 25,895 individuals who met the inclusion criteria were included in this investigation. The average age was 49.00 ± 18.45 years. Among them, 2334 participants were in the depressive symptoms group, accounting for 9.01% of the total number. Table 1 shows the comparison results of the characteristics between the depressive symptoms group (PHQ-9 ≥ 10) and the non-depressive symptoms group (PHQ-9 < 10).

Between with and without depressive symptoms groups, the latter was more likely to have a lower education level, higher BMI, lower PIR, lower occupational physical activity, lower recreational physical activity, lower vitamin C intake, lower vitamin C vegetable source intake, and lower total energy intake. In addition, for women, smokers, and stroke, hypertension, and diabetes patients, the proportion of the depressive symptoms group was significantly higher than that of the non-depressive symptoms group.

Supplementary Table S2 shows the comparison results of the characteristic between the high (>50 mg/day) and low (≤50 mg/day) vitamin C intake from vegetables. Between the groups of high and low vitamin C intake from vegetables, the latter was more likely to have a lower education level, lower PIR, lower recreational physical activity, and lower total energy intake. In addition, for women and smokers, the proportion in the low vitamin C intake from the vegetable group was significantly higher than that in the high vitamin C intake from the vegetable group.

### 3.2. Relationship between Dietary Vitamin C Intake and the Risk of Depressive Symptoms

The results are shown in Table 2. After weighted calculation, the univariate logistic regression model shows that higher dietary vitamin C intake was associated with a lower risk of depressive symptoms (*p* < 0.001). Compared with T1, the ORs (95% confidence interval) of dietary vitamin C intake T2 and T3 were 0.58 (0.50–0.68) and 0.55 (0.48–0.63).

In model 1, higher dietary vitamin C intake was related to a lower risk of depressive symptoms, and the correlation was statistically significant (*p* < 0.001). Compared with T1, the ORs (95% confidence interval) of dietary vitamin C intake T2 and T3 were 0.58 (0.50–0.68) and 0.56 (0.49–0.65), respectively. In model 2, the negative correlation between dietary vitamin C intake and depressive symptoms remained stable. Compared with T1, T2 and T3 of dietary vitamin C intake were negatively correlated with depressive symptoms, with ORs (95% confidence interval) of 0.69 (0.58–0.83) and 0.73 (0.58–0.91), respectively. The joint test of the effect for the multiple categorical variables was used, and dietary vitamin C intake was negatively correlated with depressive symptoms, with an OR value of 0.998, *p* = 0.018. In a sensitivity analysis, after excluding 2415 participants who took antidepressant medication, the association of dietary vitamin C intake with depressive symptoms was still significant in T2. Compared with T1, dietary vitamin C intake was negatively correlated with depressive symptoms, with an OR (95% confidence interval) of 0.72 (0.58–0.90).

Table 3 shows the correlation between vitamin C intake and depressive symptoms after sex stratification. In a multiple-adjusted model, the T2 and T3 groups of female dietary vitamin C intake were negatively correlated with depressive symptoms, and the ORs (95% confidence interval) were 0.613 (0.48–0.78) and 0.648 (0.48–0.87), respectively.

Table 4 shows the results of an age stratification analysis of the relationship between vitamin C intake and depressive symptoms risk. T2 in the age group 18–39 years old was inversely correlated with the risk of depressive symptoms, with an OR (95% confidence interval) of 0.708 (0.51–0.98). T2 and T3 were inversely correlated with the risk of depressive symptoms in the age group 40–59 years old, with ORs (95% confidence interval) of 0.630 (0.48–0.83) and 0.636 (0.44–0.91).

**Figure 2:**
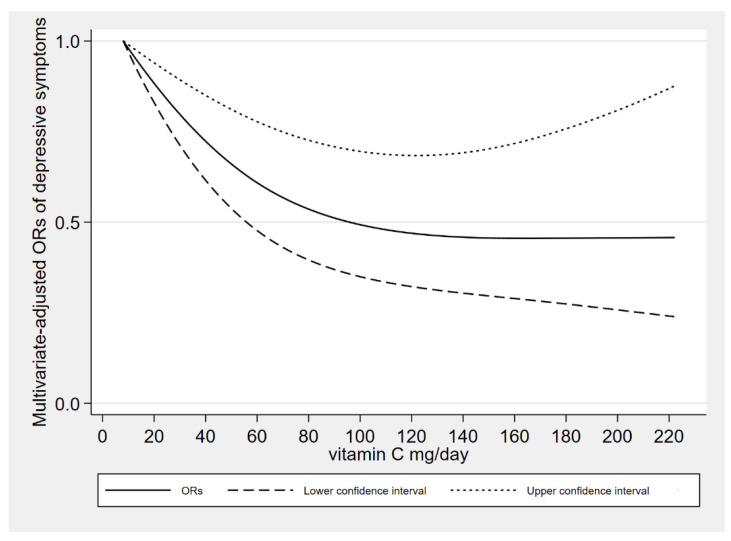
The results of dose response between dietary vitamin C intake and depressive symptoms.

In dose-response relationships, the adjustment of covariates was consistent with model 2. The dietary vitamin C intake was nonlinearly negatively associated with depressive symptoms (*P*_-nonlinearity_ = 0.009). We found an L-shaped association. The prevalence of depressive symptoms reached a plateau when the dietary vitamin C intake was higher than 126 mg/day. Figure 2 shows the dose response relationship.

### 3.3. Relationships between Dietary Vitamin C Intake Derived from Different Sources (Vegetables, Fruits), Vitamin C Supplements, Total Vitamin C Intake (Food + Supplements), and the Risk of Depressive Symptoms

The association of dietary vitamin C intake derived from vegetables and fruits with depressive symptoms risk is shown in Table 5. In a multiple-adjusted model, compared with T1, the OR (95% confidence interval) of dietary vitamin C intake derived from vegetables T3 was 0.73 (0.56–0.95). The joint test of the effect for the multiple categorical variables was used, and the dietary vitamin C intake derived from vegetables was negatively correlated with depression, with an OR value of 0.996, *p* = 0.026.

**Figure 3:**
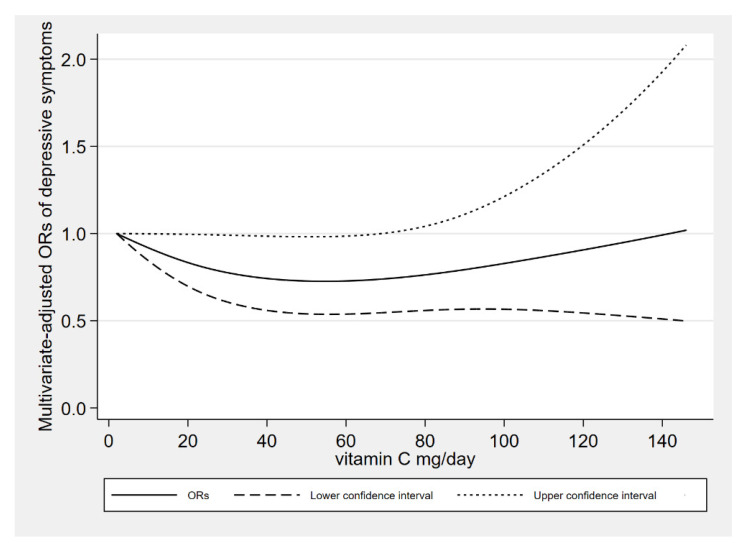
The results of dose response between dietary vitamin C intake derived from vegetables and depressive symptoms.

There was a linear association between the dietary vitamin C intake derived from vegetables and depressive symptoms (*P*_-nonlinearity_ = 0.105). When dietary vitamin C derived from vegetables was about 50 mg/day, the risk of depressive symptoms reached a relatively low level. When dietary vitamin C derived from vegetables was more than 70 mg/day, the relationships were no longer significant. The dose response between vitamin C derived from vegetables and depressive symptoms risk is shown in Figure 3.

Among 25,895 individuals, 8132 were vitamin C supplement users, accounting for 31.40% of the total participants. In the multiple adjustment model, compared with non-supplement users, vitamin C supplement users were inversely correlated with depressive symptoms risk, with an OR (95% confidence interval) of 0.78 (0.66–0.93). The association of vitamin C supplement users and non-supplement users with depressive symptoms risk is shown in Table 6.

In the multiple adjustment model, compared with T1, T2 and T3 of vitamin C intake (food + supplements) were negatively correlated with depressive symptoms, with ORs (95% confidence interval) of 0.78 (0.65–0.94) and 0.72 (0.55–0.93), respectively. The association of total vitamin C intake (food + supplements) with depressive symptoms risk is shown in Table 7.

## 4. Discussion

We found that dietary vitamin C, total vitamin C intake (food + supplements), and vitamin C derived from vegetables were negatively correlated with the risk of depressive symptoms in the general population. In stratified analyses by sex, dietary vitamin C intake was negatively correlated with the risk of depressive symptoms in female. In age stratified analyses, we discovered a negative association in the 18–39 and 40–59 year-old groups. We found that there was a nonlinear association of dietary vitamin C intake and linear association of dietary vitamin C intake derived from vegetables with the risk of depressive symptoms.

The relationship between ascorbic acid and depression was initially based on the observation of clinical manifestations of ascorbic acid deficiency [30], and some clinical studies had also explored vitamin C as an adjuvant therapy for depression [31,32]. Some studies explored the relationship between vitamin C and the emotional state in acutely hospitalized patients [31] and type 2 diabetic patients [33]; the study found that vitamin C supplementation improved their emotional levels. Some controlled experiments found that supplementing foods with high vitamin C can improve the mood of adult men [34], and the intake of vitamin C in the diet of depressed students decreased significantly [35]. In addition, some reviews summarized the existing evidence for the treatment of depression with ascorbic acid [36,37,38] and the relationship between vitamin C deficiency and depression [39,40]. However, it focused more on clinical trials, and lacked research on the relationship between dietary vitamin C intake and depressive symptoms in the general population.

A cross-sectional survey of 139 male participants aged between 18 and 35 [14] found a reversed association between dietary vitamin C status and depression. This was consistent with our results. A cross-sectional study of 279 adults aged 65 to 75 [15] found that vitamin C intake was correlated with the alleviation of depressive symptoms in community-dwelling elderly persons in Japan. It was only statistically significant in men, while our results were only statistically significant in women by sex stratification. A study of 73 residents over 60 years old found that there was no relationship between the intake of vitamin C and the improvement of depressive symptoms in individuals over 60 years old [17]. Our results also found that there was no correlation between individuals over 60 years old. Inconsistent research results may be due to the different number, age, sex, and nationality of participants.

Vitamin C may be associated with depression through the following mechanisms. Firstly, vitamin C may play an antidepressant role through its antioxidant and anti-inflammatory properties [41]. Studies have shown that vitamin C can be used as an antioxidant at low doses and as a pre-oxidant at high doses [42]. In addition, vitamin C is essential for the synthesis of the monoamine neurotransmitter dopamine, norepinephrine, and serotonin [43], and studies have found that deficiencies and disorders of these substances can lead to depression [44].

In the results of age stratification, we did not find the relationship between dietary vitamin C intake and depressive symptoms in participants over 60 years old. Nutritional requirements may change with age, and the digestive organ function of the elderly gradually declines with aging [45]. Changes caused by aging on the digestive organs of the gastrointestinal tract may affect the absorption of vitamins [46]. The oxidative damage in the elderly is aggravated. Given that the activity of antioxidant enzymes decreases with age, it is very important to provide sufficient dietary antioxidants [47].

Almost all countries encourage the consumption of fruits and vegetables [48]. According to the 2020–2025 dietary guidelines for American residents [49], the recommended dietary allowance of vitamin C for men over 18 years old is 90 mg/day, and 75 mg/day for women. According to our data, 15,828 (61.12%) participants were below the recommended dietary allowance. So, we suggest that the intake of vegetables in the daily diet should be appropriately increased to prevent chronic diseases such as depression.

There are several advantages in our research. Firstly, the dose-response relationship between vitamin C intake and depressive symptoms was discussed. Secondly, in multivariate analysis, we adjusted dietary energy intake and other confounding factors. How each covariate affects the depressive symptoms outcome was shown in Supplementary Figure S1. Thirdly, this study investigated the association between sex and age stratification. Fourthly, we also analyzed the association of vegetable-derived and fruit-derived vitamin C with the risk of depressive symptoms. Finally, because of the large sample size, the results are more reliable.

There are also several limitations in our research. First of all, the study design was cross-sectional, so it was difficult to determine the causal relationship. Secondly, PHQ-9 is a kind of screening tool that might suffer from wrong classification bias. Thirdly, a 24-h dietary recall might lead to memory bias, which might lead to an overestimation or underestimation of the results in our study. However, we used the average of two 24-h dietary recollections as the dietary vitamin C intake, which might partially reduce the recollection bias. Fourthly, cooking often destroys vitamin C in vegetables. We have not controlled the influence of cooking on vitamin C derived from vegetables. Fifth, although vegetables contain high levels of vitamin C, they also contain reasonable levels of other important micronutrients that may help improve mood, such as B vitamins, magnesium, iron, vitamin A, and so on [50]. There is also a possibility that other vitamins and minerals in vegetables may increase the health benefits of vitamin C. Sixth, the best indicator of vitamin C status is plasma ascorbic acid. Due to the limitation of data, we only explored the dietary data. Seventh, due to the limitation of data, the full race classification was not available until 2011, so we cannot analyze the relationship between different races and depression.

## 5. Conclusions

Dietary vitamin C intake and vitamin C intake derived from vegetables were inversely associated with the risk of depressive symptoms among the general population. For the results of the stratified analysis, we found it in women and 18–59 years old group. We suggest increasing the intake of vegetables in our daily diet.

## Figures and Tables

**Table 1:** Baseline characteristics of participants by depressive symptoms, NHANES 2007–2018 (*N* = 25,895).

	Non-Depressive Symptoms (PHQ < 10)	Depressive Symptoms (PHQ ≥ 10)	*p* Value
Number of participants (%) ^a^	23,561 (90.99)	2334 (9.01)	
Age (year) ^a^			<0.001
18–39	8115 (34.44)	745 (31.92)	
40–59	7332 (31.12)	906 (38.82)	
≥60	8114 (34.44)	683 (29.26)	
sex (%) ^a^			<0.001
Male	11,831 (50.21)	849 (36.38)	
Female	11,730 (49.79)	1485 (63.62)	
Race/ethnicity (%) ^a^			<0.001
Mexican American	3442 (14.61)	340 (14.57)	
Other Hispanic	2309 (9.80)	321 (13.75)	
Non-Hispanic White	10,099 (42.86)	994 (42.59)	
Non-Hispanic Black	5138 (21.81)	501 (21.47)	
Other races	2573 (10.92)	178 (7.63)	
Educational level (%) ^a^			<0.001
<high school	4946 (21.64)	748 (33.17)	
High school	12,024 (52.60)	1237 (54.86)	
>high school	5890 (25.77)	270 (11.97)	
Material status (%) ^a^			<0.001
Married/living with partner	14,981 (64.70)	1157 (50.52)	
Widowed/divorced/separated/never married	8174 (35.30)	1133 (49.48)	
Poverty–income ratio (%) ^a^			<0.001
≤0.99	4205 (17.85)	783 (33.55)	
≥1	19,356 (82.15)	1551 (66.45)	
Body mass index (%) ^a^			<0.001
<25 kg/m^2^	6768 (28.99)	541 (23.51)	
25 to <30 kg/m^2^	7749 (33.19)	589 (25.60)	
≥30 kg/m^2^	8831 (37.82)	1171 (50.89)	
Work activity (%) ^a^			0.003
Vigorous	4643 (19.72)	464 (19.91)	
Moderate	5232 (22.22)	452 (19.39)	
Other	13,675 (58.07)	1415 (60.70)	
Recreational activity (%) ^a^			<0.001
Vigorous	5710 (24.20)	259 (11.10)	
Moderate	6241 (26.49)	457 (19.58)	
Other	11,665 (49.27)	1618 (69.32)	
Alcohol consumption (%) ^a^	17,117 (73.94)	1743 (75.55)	0.182
Smoke at least 100 cigarettes in life (%) ^a^	9710 (42.34)	1342 (58.42)	<0.001
stroke ^a^	775 (3.47)	178 (7.93)	<0.001
Diabetes (%) ^a^	2954 (12.54)	475 (20.35)	<0.001
Hypertension (%) ^a^	12,708 (53.94)	1410 (60.41)	<0.001
Vitamin C intake (mg/d) ^b^	64.40 (83.45)	47.13 (76.1)	<0.001
Total energy (kcal/d) ^b^	1898.5 (982.5)	1796.25 (1005.5)	<0.001
Vitamin C from vegetable sources (mg/d) ^c^	23.25 (40.65)	18.1 (31.05)	<0.001
Vitamin C from fruit source (mg/d) ^c^	48.55 (71.9)	46.05 (70.9)	0.140
Vitamin C supplements (mg/d) ^c^	90 (190)	70 (167)	0.037

Data are number of participants (weighted percentage) or medians (interquartile ranges). PHQ, Patient Health Questionnaire. ^a^ Chi-square test was used to compare the percentage between participants with and without depressive symptoms. ^b^ Mann-Whitney U test was used to compare the difference between participants with and without depressive symptoms. ^c^ Among 25,895 individuals, 18,341 had complete vitamin C data from vegetable sources, 10,700 had complete vitamin C data from fruit sources, and 8132 were vitamin C supplement users.

**Table 2:** Weighted ORs and 95% CIs for depressive symptoms according to terciles of dietary vitamin C intake.

	Cases/Participants	Crude	Model 1 ^a^	Model 2 ^b^
Vitamin C intake (mg/d)				
T1 (<39.55)	1049/8642	1.00 (ref)	1.00 (ref)	1.00 (ref)
T2 (39.55 to 92.95)	670/8623	0.58 (0.50–0.68) **	0.58 (0.50–0.68) **	0.69 (0.58–0.83) **
T3 (>92.95)	615/8630	0.55 (0.48–0.63) **	0.56 (0.49–0.65) **	0.73 (0.58–0.91) *

^a^ Adjusted for age, and sex. ^b^ Adjusted for age, sex, race, educational level, marital status, body mass index, work physical activity, recreational physical activity, ratio of family income to poverty, smoking status, alcohol consumption, energy (continuous), hypertension, diabetes, stroke, time, and time*vitamin C. * *p* < 0.05. ** *p* < 0.001.

**Table 3:** Association between dietary vitamin C intake and depressive symptoms after sex stratification.

Dietary Vitamin C Intake	Odds Ratio	95%CI	*p* Value
Male			
T1 (<39.55)	1	1	
T2 (39.55 to 92.95)	0.833	0.63–1.11	0.205
T3 (>92.95)	0.885	0.61–1.29	0.523
Female			
T1 (<39.55)	1	1	
T2 (39.55 to 92.95)	0.613	0.48–0.78	*p* < 0.001
T3 (>92.95)	0.648	0.48–0.87	0.004

**Table 4:** Association between dietary vitamin C intake and depressive symptoms after age stratification.

Dietary Vitamin C Intake	Odds Ratio	95%CI	*p* Value
18–39 years old			
T1 (<39.55)	1	1	
T2 (39.55 to 92.95)	0.708	0.51–0.98	0.039
T3 (>92.95)	0.844	0.58–1.23	0.371
40–59 years old			
T1 (<39.55)	1	1	
T2 (39.55 to 92.95)	0.630	0.48–0.83	0.003
T3 (>92.95)	0.636	0.44–0.91	0.014
≥60 years old			
T1 (<39.55)	1	1	
T2 (39.55 to 92.95)	0.849	0.58–1.24	0.393
T3 (>92.95)	0.866	0.48–1.57	0.633

**Table 5:** Association between vitamin C intake derived from vegetables and fruits with depressive symptoms.

KERRYPNX	Cases/Participants	Crude	Model ^a^	Model ^b^
vitamin C derived from vegetables (mg/d)				
T1 (<13.8)	606/6118	1.00 (ref)	1.00 (ref)	1.00 (ref)
T2 (13.8 to 37.75)	510/6114	0.81 (0.66–0.98) *	0.82 (0.67–1.00)	0.89 (0.72–1.09)
T3 (>37.75)	383/6109	0.64 (0.53–0.77) **	0.66 (0.54–0.79) **	0.73 (0.56–0.95) *
vitamin C derived from fruits (mg/d)				
T1 (<26.55)	253/3567	1.00 (ref)	1.00 (ref)	1.00 (ref)
T2 (26.55 to 73.9)	221/3568	0.88 (0.66–1.19)	0.89 (0.66–1.20)	0.93 (0.69–1.26)
T3 (>73.9)	219/3565	0.87 (0.65–1.17)	0.92 (0.68–1.24)	0.97 (0.68–1.40)

^a^ Adjusted for age, and sex. ^b^ Adjusted for age, sex, race, educational level, marital status, body mass index, work physical activity, recreational physical activity, ratio of family income to poverty, smoking status, alcohol consumption, energy (continuous), hypertension, diabetes, stroke, time and time*year. * *p* < 0.05. ** *p* < 0.001.

**Table 6:** Association between vitamin C supplement users and non-supplement users with depressive symptoms.

	Cases/Participants	Crude	Model ^a^	Model ^b^
non-supplement users	1755/17,763	1.00 (ref)	1.00 (ref)	1.00 (ref)
supplement users	579/8132	0.60 (0.52–0.70) **	0.59 (0.51–0.70) **	0.78 (0.66–0.93) *

^a^ Adjusted for age, and sex. ^b^ Adjusted for age, sex, race, educational level, marital status, body mass index, work physical activity, recreational physical activity, Ratio of family income to poverty, smoking status, alcohol consumption, energy (continuous), hypertension, diabetes, stroke, time and time*year. * *p* < 0.05. ** *p* < 0.001.

**Table 7:** Association between total vitamin C intake (food + supplements) and depressive symptoms.

	Cases/Participants	Crude	Model 1 ^a^	Model 2 ^b^
Vitamin C intake (food + supplements) (mg/d)				
T1 (<53.25)	1024/8634	1.00 (ref)	1.00 (ref)	1.00 (ref)
T2 (53.25 to 132.9)	723/8630	0.65 (0.56–0.75) **	0.65 (0.56–0.76) **	0.78 (0.65–0.94) *
T3 (>132.9)	587/8631	0.50 (0.43–0.58) **	0.51 (0.44–0.60) **	0.72 (0.55–0.93) *

^a^ Adjusted for age, and sex. ^b^ Adjusted for age, sex, race, educational level, marital status, body mass index, work physical activity, recreational physical activity, Ratio of family income to poverty, smoking status, alcohol consumption, energy (continuous), hypertension, diabetes, stroke, time and time*year. * *p* < 0.05. ** *p* < 0.001.

## Data Availability

The data is contained within the article or supplementary material.

## Supplementary Materials

The following are available online at https://www.mdpi.com/article/10.3390/antiox10121984/s1, Figure S1: How each covariate affects the depressive symptoms outcome. Table S1: The classifications of covariates, Table S2: The comparison results of the characteristic between the high (>50 mg/day) and low (≤50 mg/day) vitamin C intake from vegetables.
