# Supplementary material for: Dietary Vitamin C and Vitamin C Derived from Vegetables Are Inversely Associated with the Risk of Depressive Symptoms among the General Population
Source: Antioxidants (Basel). 2021 Dec 13;10(12):1984. doi: 10.3390/antiox10121984 (PMC8750333; doi:10.3390/antiox10121984)
Supplement: Supplementary file 1 [file antioxidants-10-01984-s001.zip › antioxidants-1437292-table S1.pdf]

Table S1. The classifications of covariates.

Covariates	Classifications
Gender	Male; Female
Age (year)	18-39; 40-59; ≥60
Educational level	Below high school; High school; Above high school
Marital status	Married/living with partner; Widowed/divorced/separated/ Never married/
Poverty-income ratio	≤0.99; and ≥1
Race	Mexican American; Other Hispanic; Non-Hispanic White; Non-Hispanic Black; Other race
Body mass index (BMI)	≤25 kg/m2; 25 to <30 kg/m2; ≥30 kg/m2
Smoked at least 100 cigarettes in life	No; Yes
Had at least 12 alcohol drinks/year	No; Yes
Occupational physical activity	Vigorous; Moderate; Other
Recreational physical activity	Vigorous; Moderate; Other
Hypertension [a]	No; Yes
Diabetes [b]	No; Yes
Stroke [c]	No; Yes
Total energy intake (kcal/d) [d]	Continuous

[a] Systolic blood pressure (SBP) ≥130 mm Hg, or diastolic blood pressure (DBP) ≥80 mm Hg, or currently taking antihypertensive drugs were hypertensive patients. [b] Diabetes was defined as self-reported physician diagnosis or taking insulin, or anti-diabetic pills. [c] Stroke was defined as self-reported physician diagnosis of stroke. [d] Total energy intake of each participant was the average of two 24-hour dietary energy intake.
