# Supplementary material for: Dietary Vitamin C and Vitamin C Derived from Vegetables Are Inversely Associated with the Risk of Depressive Symptoms among the General Population
Source: Antioxidants (Basel). 2021 Dec 13;10(12):1984. doi: 10.3390/antiox10121984 (PMC8750333; doi:10.3390/antiox10121984)
Supplement: Supplementary file 1 [file antioxidants-10-01984-s001.zip › antioxidants-1437292-table S2.pdf]

Table S2. The characteristics of participants with high or low vitamin C intake from vegetables

	**Low Vitamin C Intake from Vegetables (≤50mg/day)**	**High Vitamin C Intake from Vegetables (>50mg/day)**	***p* Value**
Number of participants (%) [a]	13735 (74.89)	4606 (25.11)	
			0.0029
Age (year) [a]	4592 (33.43)	1305 (28.33)	
18-39	4370 (31.82)	1501 (32.59)	
40-59	4773 (34.75)	1800 (39.08)	
≥60			0.0288
Gender (%) [a]	6524 (47.50)	2302 (49.98)	
Male	7211 (52.50)	2304 (50.02)	
Female			0.0036
Race/ethnicity (%) [a]	1919 (13.97)	588 (12.77)	
Mexican American	1281 (9.33)	417 (9.05)	
Other Hispanic	6209 (45.21)	1872 (40.64)	
Non-Hispanic White	3026 (22.03)	976 (21.19)	
Non-Hispanic Black	1300 (9.46)	753 (16.35)	
Other races			< 0.001
Educational level (%) [a]	2888 (21.68)	690 (15.50)	
< high school	7219 (54.19)	2224 (49.94)	
High school	3214 (24.13)	1539 (34.56)	
> high school			0.3333
Material status (%) [a]	8684 (64.32)	2943 (64.95)	
Married/living with partner	4817 (35.6)	1588 (35.05)	
Widowed/divorced/separated/never married			
Poverty–income ratio (%) [a]			0.0021
≤0.99	11223 (81.71)	3958 (85.93)	
≥1	2512 (18.29)	648 (14.07)	
Body mass index (%) [a]			0.1725
< 25 kg/m2	3810 (28.00)	1368 (29.95)	
25 to <30 kg/m2	4437 (32.61)	1517 (33.22)	
≥30 kg/m2	5359 (39.39)	1682 (36.83)	
Work activity (%) [a]			0.1997
Vigorous	2668 (19.43)	929 (20.18)	
Moderate	3085 (22.47)	1056 (22.94)	
Other	7975 (58.09)	2618 (56.88)	
Recreational activity (%) [a]			< 0.001
Vigorous	3024 (22.02)	1360 (29.53)	
Moderate	3642 (26.52)	1251 (27.17)	
Other	7066 (51.46)	1994 (43.30)	
Alcohol consumption (%) [a]	9895 (73.29)	3741 (81.66)	< 0.001
Smoke at least 100 cigarettes in life (%) [a]	5835 (43.57)	1803 (39.43)	0.0002
stroke [a]			
Diabetes (%) [a]	523 (4.00)	159 (3.55)	0.2668
Hypertension (%) [a]	1866 (13.59)	635 (13.79)	0.5193
Total energy (kcal/d) [b]	7554 (55.00)	2627 (57.03)	0.3944
	1905 (964.5)	2009 (1039)	< 0.001
