# Supplementary figures and images for: Dietary Vitamin C and Vitamin C Derived from Vegetables Are Inversely Associated with the Risk of Depressive Symptoms among the General Population
Source: Antioxidants (Basel). 2021 Dec 13;10(12):1984. doi: 10.3390/antiox10121984 (PMC8750333; doi:10.3390/antiox10121984)

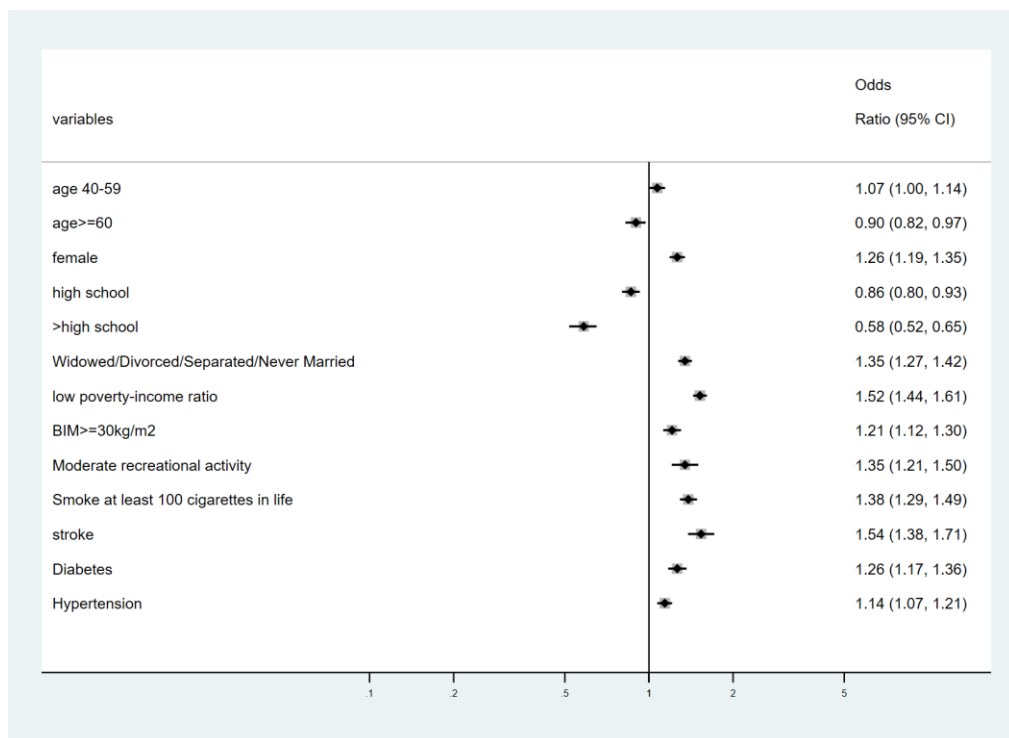

Figure S1: How each covariate affects the depressive symptoms outcome.

Supplement: Supplementary file 1 [file antioxidants-10-01984-s001.zip › antioxidants-1437292-figure S1.pdf]
